# Enhanced discovery of bacterial laccase‐like multicopper oxidase through computer simulation and metagenomic analysis of industrial wastewater

**DOI:** 10.1002/2211-5463.70037

**Published:** 2025-04-15

**Authors:** Ting Cui, Ariel Kushmaro, Hana Barak, Anja Poehlein, Rolf Daniel, Hans‐Jürgen Mägert

**Affiliations:** ^1^ Anhalt University of Applied Sciences Köthen Germany; ^2^ Avram and Stella Goldstein‐Goren Department of Biotechnology Engineering Ben‐Gurion University of the Negev Beer‐Sheva Israel; ^3^ Genomic and Applied Microbiology & Göttingen Genomics Laboratory, Institute of Microbiology and Genetics Georg‐August University of Göttingen Germany

**Keywords:** computer simulation, laccase discovery, laccase‐like, metagenomics, multicopper oxidase

## Abstract

Laccases belong to the superfamily of multicopper oxidases (MCO), a group of enzymes with the ability to reduce oxygen to water in a reaction without producing harmful byproducts. Laccase activity is influenced by many factors, such as structure; the number, location and binding status of copper ions; and the substrate‐binding status. A large number of sequences that have not been experimentally characterized yet have been annotated as laccases. However, the biological functions of the characterized MCOs are considered to vary, and the substrate spectrum overlaps with that of other MCOs. Here, we identified 34 putative bacterial laccase sequences from metagenome data for industrial wastewater. We used machine‐learning tools to screen enzymes with laccase activity by combining the T1 copper‐binding capacity, the overall copper‐binding capacity and the substrate‐binding capacity. We also used the software comparisons to remove sequences with large discrepancies between different software applications. Three‐dimensional structures of identified enzymes were predicted using alphafold, the positions of metal ions within the proteins were predicted by metal3d and autodock‐vina, and their docking with ABTS [i.e. 2,2′‐azinobis(3‑ethylbenzo‐6‑thiazolinesulfonic acid)] as a substrate was predicted by rosetta and autodock‐vina. Based on the docking results, we selected 10 high‐scoring proteins, two low‐scoring proteins and one composite protein for expression using the pET‐21d (+) vector. In line with our predictions, all selected high‐scoring proteins exhibited activity towards ABTS. Overall, we describe a method for discovering and designing novel bacterial laccase‐like multicopper oxidases, offering increased possibilities for the degradation of various harmful components derived from environmental pollution.

## Graphical abstract

We obtained potential bacterial laccase‐like multicopper oxidase (LMCO) sequences through metagenomic sequencing. All sequences exhibited significant differences from known LMCOs in databases. To select the most promising candidates, we performed structure prediction and molecular docking using alphafold2, metal3d and rosetta. Ultimately, all selected proteins identified with laccase activity.

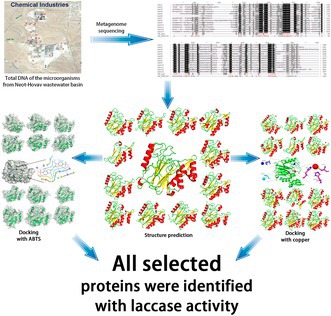

**Abbreviations**
ABTS: 2,2′‐azinobis(3‑ethylbenzo‐6‑thiazolinesulfonic acid)
DUF152: domain of unknown function 152
IPTG: isopropyl thio‐β‐d‐galactoside
LMCO: laccase‐like multicopper oxidases
MCO: multicopper oxidases

Discovered in 1883, laccases belong to the superfamily of multicopper oxidases (MCO), a group of enzymes with diverse biological functions and the ability to reduce oxygen to water in a reaction without producing harmful byproducts [1, 2, 3]. For example, poly‐copper oxidases and laccases have been found in fungi, higher plants, bacteria and insects. They are involved in lignin biodegradation, pigment production and plant pathogenesis. In plants, they have a potential function in the biosynthesis of lignin [2]. Most of the laccases that have been studied and used in industrial processes are of fungal origin and generally 60–100 kDa in size. Laccases from eukaryotes tend to be glycosylated [4]. They use molecular oxygen to oxidize a broad range of aromatic and non‐aromatic compounds through a free‐radical‐catalyzed reaction mechanism, often involved in the process of lignin degradation [2, 5]. MCOs with laccase activity in prokaryotes are often described as ‘polyphenol oxidases’, ‘multi‐copper oxidases’ or ‘laccase‐like enzymes’. The enzymes in the MCO family have similar molecular structures and also similar catalytic functions and overlapping substrate profiles. They are divided into four important groups: laccase (EC1.10.3.2), ascorbate oxidase (EC1.10.3.3), ferroxidase (EC1.16.3.1) and nitrite reductase (EC1.7.2.1) [6]. They all catalyze the oxidation of typical laccase substrates. The MCOs of bacteria with laccase activity always have multiple functions [7]. Most naturally expressed bacterial laccases are intracellular [8]. Furthermore, most of the identified bacterial laccases have not been reported to be directly involved in lignin degradation [9, 10]. In bacteria, they have been reported to play a role in melanogenesis, spore shell resistance, morphogenesis and copper detoxification [11, 12]. The optimal reaction temperature for most of the fungal laccase enzymes ranges from 25 to 50 °C [1, 13]; however, bacterial laccases typically have relatively high optimal reaction temperatures, thus increasing the breadth of laccase applications [14].

The amino acid sequences of the laccases exhibit low identity to each other, but their three‐dimensional structures are similar [5]. The initial electron acceptor in the redox reaction catalyzed by laccase is the T1 copper ion, which is located in a pocket close to the enzyme surface. Bacterial laccases have a low redox potential, whereas high‐redox‐potential laccases are widely found in fungi [9, 10]. The reduction of T1 copper ions is the rate‐limiting step in the laccase‐catalyzed reaction, and binding site of the T1 copper ions is also the focus of laccase screening and design [15, 16]. Phenolic compounds are oxidized by laccase to phenoxy radicals. However, the reaction is reversible, based on the stability of phenoxy radicals [17]. Redox cycling of phenolic substrates as mediators broadens the range of laccase substrates [18, 19]. The high non‐specific oxidative capacity of laccases and the fact that molecular oxygen acts as an electron acceptor make them excellent candidates for industrial and biological applications [20]. Laccases have many potential applications, such as in organic chemistry, biofuel production and bioremediation, as well as in the food, paper, textile, furniture, construction, paint, cosmetic and biomedical industries [21, 22, 23].

Typical laccases have four copper ligands and a molecular mass in the range 50–70 kDa or greater, whereas atypical laccases have a molecular mass between 20 and 40 kDa, with zinc substituting more often for the copper ion [15]. The domain of unknown function 152 (DUF152) family of laccase enzymes, such as RL5, BT4389 and YfiH, exhibit activity in the oxidation of aromatic compounds. RL5 has been shown by mass spectrometry to still contain four copper atoms per molecule of enzyme [24, 25]. The molecular masses of the 11 active laccases obtained in this study ranged from 24 to 28.5 kDa. A large number of sequences that have not yet been experimentally characterized have been annotated as laccase. However, the biological functions of the characterized MCOs are assumed to vary, and the substrate spectrum overlaps with that of other MCOs [6]. The enzymes in this study are called laccase‐like multicopper oxidase (LMCO).

As a result of advances in sequencing and information technology, metagenomics has generated a huge amount of sequencing data, thus allowing us to obtain a glimpse of the potential of uncultured microorganisms and search for new LMCOs [26, 27]. However, the accuracy of traditional functional annotation needs to be improved, and experimental identification is both time consuming and labor intensive. Modeling and analysis of protein sequences through new information technologies and machine learning will pave the way forward. Four technologies, namely alphafold, metal3d, rosetta and autodock‐vina, represent excellent tools in the search for novel LMCOs. alphafold allows the prediction of protein structures with atomic precision, even when similar structures are not known [28, 29]. Using a deep learning algorithm, the error of alphafold2 is less than the width of an atom [30]. metal3d predicts the location of metal ions in proteins and outputs a confidence measure for each predicted site; it is also applicable to proteins with few orthologs in the protein database [31]. autodock‐vina quickly enables molecular docking and virtual screening on multicore CPUs [32]. In addition, as an early software for predicting protein–small molecule interactions, rosettaLigand has been improved since 2006 to allow flexibility in receptor backbones and greater flexibility in ligands, and rosetta‐highresdocker has further increased usage flexibility [33, 34]. Interface_delta_X in the rosetta output represents the difference between the bound protein–ligand complex and the unbound protein and ligand with rosetta Energy Units. These tools have greatly improved the accuracy and efficiency of the discovery of novel LMCOs.

In the present study, (a) the sequences of LMCOs potentially present in industrial wastewater were searched, using metagenome sequencing data as the starting point. (b) Prediction of three‐dimensional structures of potential LMCOs was performed using alphafold2. (c) Predictions of metal ligand positions with confidence metric or affinities were made using metal3d and autodock‐vina. (d) Predictions of protein affinities to substrates such as 2,2′‐azinobis(3‑ethylbenzo‐6‑thiazolinesulfonic acid) (ABTS) were made using rosetta‐highresdocker and autodock‐vina. (e) Using the above simulation method, suitable fragments from other existing LMCOs were sought to complete an incomplete LMCO sequence. (f) In the measurement of LMCO activity, 11 active LMCOs were determined, and parameters and methods for screening LMCOs by structure prediction and analysis were validated. This study leverages the development of sequencing and information technologies to screen and design new LMCOs that meet defined needs.

## Materials and methods

### Chemicals and reagents

The total DNA of the microorganisms was isolated from the Neot‐Hovav wastewater basin in Israel, which contains industrial effluents. Wastewater from these basins is characterized by high salt concentrations of more than 160 g·L^−1^ and the presence of large amounts of toxic, halogenated organic compounds [35].

Phusion High‐Fidelity PCR Master Mix, FastDigest, T4 DNA Ligase, DNA fragments, oligonucleotide primers, B‐PER Complete Bacterial Protein Extraction Reagent, Qubit dsDNA Assay Kits and Ni‐NTA Spin Columns were purchased from Thermo Fisher Scientific (Waltham, MA, USA). (ABTS), phenol/chloroform/isoamyl alcohol and isopropyl thio‐β‐d‐galactoside (IPTG) were purchased from Merck (Darmstadt, Germany).

### Metagenome sequencing

Illumina sequencing libraries were prepared using the Illumina Nextera DNA Sample Preparation Kit and the Nextera Index Kit for multiplexing as recommended by the manufacturer (Illumina Inc., San Diego, CA, USA). To assess the quality and size of the libraries, samples were analyzed with an Agilent Bioanalyzer 2100 using an Agilent High‐Sensitivity DNA Kit as recommended by the manufacturer (Agilent Technologies, Waldbronn, Germany). Concentrations of the libraries were determined using the Qubit^®^ dsDNA HS Assay Kit in accordance with the manufacturer's instructions (Life Technologies GmbH, Darmstadt, Germany). Sequencing was performed using the HiSeq2500 instrument (Illumina Inc.), using the HiSeq^®^ Rapid SBS Kit v2 (500 Cycle) and the HiSeq^®^ Rapid PE Cluster Kit v2 for sequencing in the paired‐end mode with 2 × 250 cycles. Raw reads were quality filtered using trimmomatic v0.39 (x1) and subsequently *de novo* assembled with metaspades v3.11.1 (x2). Contigs larger than 1 kb were annotated with prokka 1.12.0 (x3) in meta mode to improve gene predictions for highly fragmented genomes [36, 37, 38].

### Three‐dimensional structure prediction

Three‐dimensional structure prediction was performed using NVIDIA Tesla T4 (https://www.nvidia.com/en‐gb/datacenter/teslat4) running alphafold2 for the 34 possible LMCO sequences given by the sequencing results. To achieve the best accuracy, all predictions work with the parameter ‘‐model_preset=mono_casp14’.

### Metal ligand position prediction

The metal ligand position prediction was performed using both metal3d and autodock‐vina. metal3d was run with the following parameters: ‘‐‐metalbinding ‐‐writeprobes ‐‐softexit’. The first four copper ions in the result with clearly distinct binding regions and their confidence metric were collected. autodock‐vina was first used to perform global docking of proteins with copper ions. Thereafter, with the four best‐scoring metal ligand positions in the results predicted by metal3d, autodock‐vina was run again for local docking centered on each of them. The affinity results were collected, filtering the results on the basis of where autodock‐vina did not contradict the metal3d results to select the best model. metal3d results, which matched the autodock results, were used; the best copper ion score was recorded; and an average of the top four copper ion scores was calculated.

### Dock for substrate

Using the ‘sdf’ file of ABTS (CID: 9570474) downloaded from PubChem to run autodock‐vina, the affinity values were counted. Using the ‘‐nstruct 50 000’ parameter centered on the first three results given by autodock‐vina, rosetta‐highresdocker was run to perform the dock, and the ‘interface_delta_X’ values were counted to select the best model.

### Mending an incomplete LMCO sequence

**Fig. 1:**
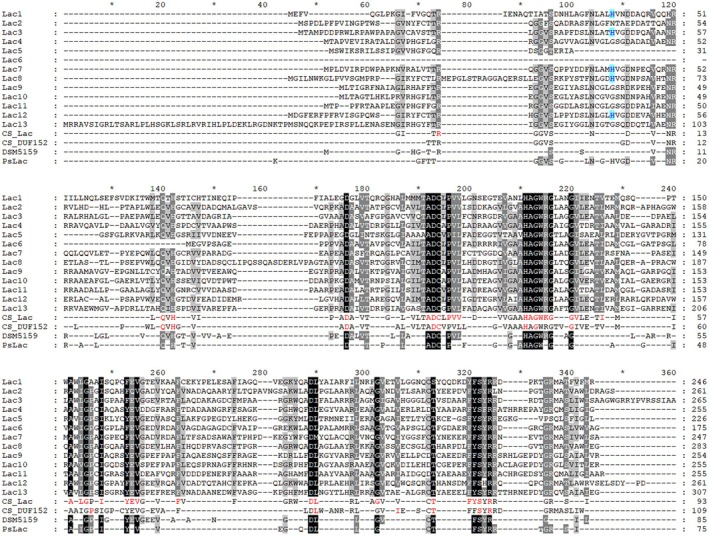
Multiple alignment of the predicted laccase‐like multicopper oxidase (LMCO) sequences in this study using geneious software with ‘clustal omega’ and genedoc software for drawing. Conserved sequences of LMCOs in this study were named CS_Lac. Conserved sequences of the DUF152 family from NCBI (pfam02578) were named Cs_DUF152 [46]. Red residues in the conserved sequence were obtained using the option Color Bits for Identity.

As shown in Fig. 1, one predicted LMCO sequence, Lac6, was found to lack a significant portion of its N‐terminal sequence. The results of prediction and docking showed that the typical complete T1 copper‐binding site (His…Cys…His) could not be formed. To address this, the sequence ‘HAGWRG’ was selected for splicing based on the conservation of T1 copper‐binding sites and structural similarity among MCOs. After sequence alignment, the N‐terminal sequences of other predicted LMCOs were used to mend Lac6. The mended sequence underwent further prediction and docking analysis, and the optimal result was selected. The DNA fragments were obtained using fusion PCR [39].

### Expression and purification

DNA sequences were generated using *Escherichia coli* codon usage and the NovoPro tool and were synthesized by Thermo Fisher Scientific. The synthetic DNA fragment was ligated into the pET‐21d(+) vector using *Nco*I and *Xho*I restriction endonucleases with T4 DNA ligase, and *E. coli* BL21(DE3) cells were transformed with the ligation products. Using LB medium with 5% glycerol and 100 μg·mL^−1^ ampicillin, the culture was incubated until D_600_ of 0.7 ± 0.05 was reached. IPTG at a final concentration of 1 mm and CuSO_4_ at a final concentration of 0.25 mm were then added, and the mixture was incubated at 37 °C for 4 h. Cultures were collected and centrifuged for 5 min at 1800 ***g***, and B‐PER Complete Bacterial Protein Extraction Reagent with 5 mL reagent·g^−1^ biomass was added to resuspend and lyse the cells. The cell fractions obtained were used to confirm successful expression by SDS/PAGE, as well as to qualitatively determine enzyme activity. The same medium and parameters were used for overnight induction at 16 °C, cultures were collected and centrifuged for 5 min at 1800 ***g***, phosphate‐buffered saline was used to resuspend the cells, and ultrasound was used to disrupt the cells. HisPur™ Ni‐NTA Spin Columns were used to purify the target proteins. The elution buffer was phosphate‐buffered saline with 250 mm imidazole. The protein concentration was determined using Rotiquant (Carl ROTH, Karlsruhe, Germany), in accordance with the manufacturer's instructions.

### Enzyme activity measurement

Cellular fractions were used to qualitatively measure enzyme activity, and LMCO kinetics were measured using purified proteins. Enzymatic reactions were assayed using a DS‐11+ Spectrophotometer (DeNovix, Wilmington, DE, USA) at 420 nm with a final concentration of 100 mm acetate buffer (CH_3_COOH with CH_3_COONa, pH 4.6) with 2.5 mm copper sulfate, using 0.5 mm ABTS (PanReac AppliChem, Chicago, IL, USA; ε 420 = 38 000 m^−1^·cm^−1^) as the substrate. BL21 (DE3) containing empty plasmid carriers induced by culture under the same conditions was used as a negative control when qualitatively measuring enzyme activity. For quantitative measurement of enzyme activity using purified protein, purified elution buffer was used as a negative control. Enzyme activities were measured at pH 3.8, 4.0, 4.2, 4.4, 4.6, 4.8, 5.0, 5.2, 5.4, 5.6 and 5.8 and at 37, 45, 50, 55, 60, 65, 70, 80 and 90 °C using the optimal pH buffer. Qualitative measurements were performed at 37 °C with pH 4.6. Michaelis–Menten kinetics was measured using ABTS at a final concentration of 10–200 μm.

Copper ions were replaced with zinc ions for qualitative activity measurements during incubation, induction and enzyme activity assays.

## Results and Discussion

### Laccase sequence analysis

We identified 34 putative LMCO sequences within the 179 233 predicted amino acid sequences obtained by prokka 1.12.0 (x3). These data derived from an initial dataset containing 48 119 assembled genomes. The LMCOs used in this study were named Lac1 to Lac13. These sequences were subjected to blast with the UniProtKB/Swiss‐Prot (reviewed) database, which is manually annotated and contains the results of the experiments, and the computer‐annotated UniProtKB/TrEMBL (unreviewed) database. As shown in Table 1, which displays the BLAST results, most of the sequences do not match the sequences in UniProtKB/Swiss‐Prot (reviewed), and most of the sequences in this study matched to sequences in UniProtKB/TrEMBL (unreviewed) also have identity below 70%. None of the matched reviewed enzymes have been characterized for laccase or MCO activity. All of the matched reviewed sequences were identified and unreviewed sequences annotated as purine nucleoside phosphorylase. It has been shown that DUF152 (YfiH) proteins have the potential to catalyze not only typical laccase substrates, but also nucleoside analogs. That is why this class of proteins is also often annotated as purine nucleoside phosphorylase. DSM 5159 from *Thermomicrobium roseum* possesses both laccase and purine nucleoside phosphorylase activities [40]. PsLac from *Peribacillus simplex* used purine nucleoside phosphorylase as a template for homology modeling and ultimately validated laccase activity [41]. Id17_09670 also found no significant matches in the database and was matched in Uniport with purine nucleoside phosphorylase. However, it was predicted as being highly likely to possess laccase activity [42].

**Table 1:** Result of Uniprot BLAST. Lac1 – Lac13 are the sequences in this study.

	UniProtKB/swiss‐Prot (reviewed)		UniProtKB/TrEMBL (unreviewed)	
	Entry	Identity	Entry	Identity
Lac1	Not found		N8VZY8	85.4%
Lac2	Not found		A0A2N4UKA8	54.7%
Lac3	P33663 [43]	62.5%	A0A6H9XVE4	66.4%
Lac4	Not found		A0A2N0H5C8	77.6%
Lac5	Q9RT03 [44]	37.2%	A0A8K0A0J6	40.6%
Lac6	P33663 [43]	57.5%	A0A091BSI1	60.7%
Lac7	A0A384KG77 [45]	54.5%	A0A090AFC5	60.1%
Lac8	Not found		A0A2U1CHW3	59.0%
Lac9	Not found		A0A839SYC3	61.3%
Lac10	Not found		A0A967F2F3	62.6%
Lac11	Not found		A0A2L0VLX7	100.0%
Lac12	A0A384KG77 [45]	47.5%	A0A1C3K029	59.3%
Lac13	Not found		A0A7W9S5D7	82.4%

Multiple sequence alignment was carried out using geneious prime software (https://www.geneious.com), as depicted in Fig. 1. The conserved sequence of the DUF152 family was derived from the NCBI Conserved Protein Domain Family (pfam02578), and the Row Display was used ‘max. 100 rows’ [46]. The identity between these sequences can be visualized directly in Table 2. These bacterial LMCOs lack the conserved HXH sequence typically used by laccases for binding copper ions and facilitating electron transfer. It is worth noting that Lac13 possesses an extended N‐terminal sequence. Additionally, a prediction of transmembrane structural domains using the TMHMM tool revealed the complete absence of such domains within the sequence [47, 48].

**Table 2:** Identity between the sequences of Lac1 to Lac13.

	Lac1	Lac2	Lac3	Lac4	Lac5	Lac6	Lac7	Lac8	Lac9	Lac10	Lac11	Lac12
Lac2	27.5%											
Lac3	30.1%	38.5%										
Lac4	23.6%	32.2%	33.1%									
Lac5	21.2%	25.4%	22.1%	23.9%								
Lac6	23.9%	35.1%	35.6%	29.8%	22.0%							
Lac7	32.0%	40.2%	46.1%	29.0%	26.0%	32.0%						
Lac8	28.7%	40.4%	40.3%	30.4%	22.2%	30.7%	38.0%					
Lac9	23.4%	34.3%	29.5%	50.6%	26.4%	28.1%	29.6%	30.3%				
Lac10	24.6%	32.8%	29.7%	50.2%	25.0%	26.7%	30.5%	31.5%	60.2%			
Lac11	24.0%	34.3%	33.5%	60.6%	26.3%	31.5%	29.5%	31.3%	48.4%	49.6%		
Lac12	31.7%	45.0%	39.1%	31.1%	23.8%	31.8%	39.4%	42.5%	30.5%	29.9%	31.6%	
Lac13	20.0%	26.5%	27.7%	42.6%	22.2%	25.9%	25.6%	27.0%	41.3%	38.2%	36.7%	25.6%

**Fig. 2:**
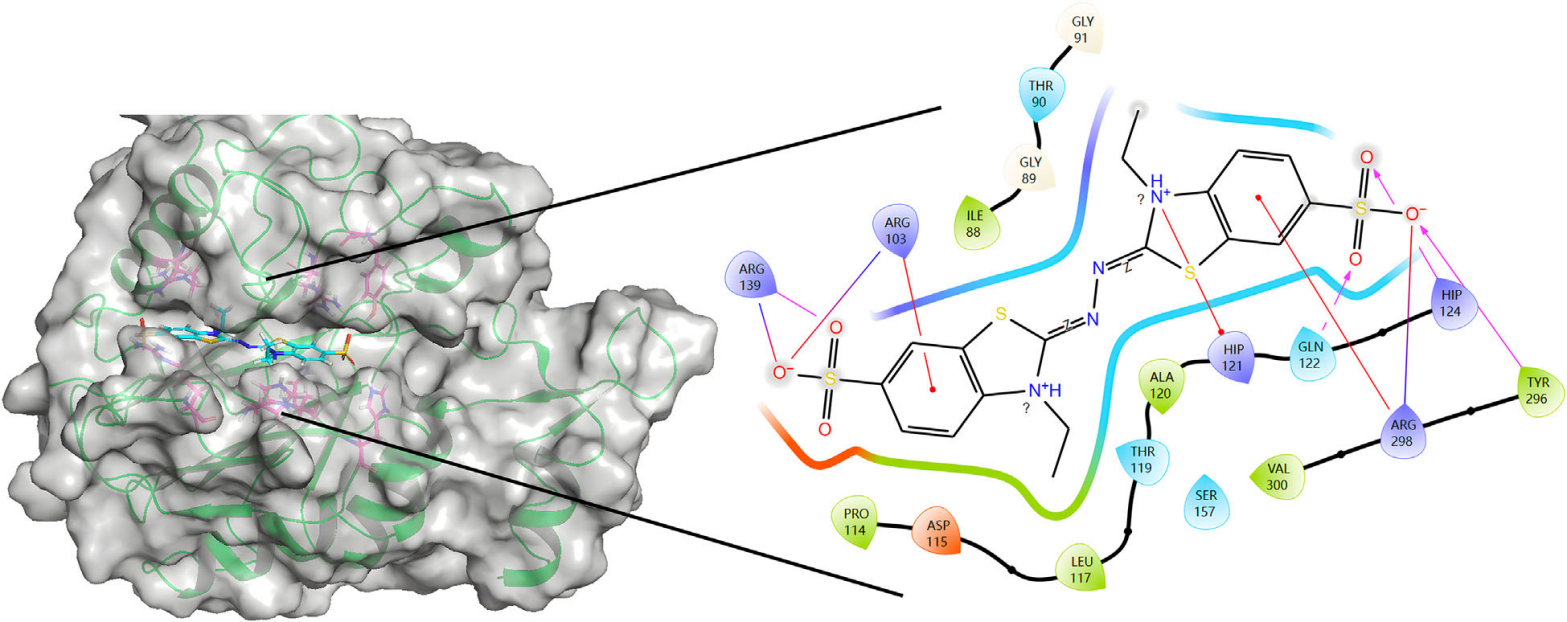
Result of Lac13–ABTS docking. Y296, Q122 and H124 form hydrogen bonds (purple arrow) with sulfonyl hydroxide on both sides of the ABTS. R103, R139 and R298 form salt bridges (purple line) with the hydroxyl groups on the sulfonic acid groups on both sides of the ABTS. R103 and R298 form Pi‐Cat interactions (red line) with the benzene rings on each side. Pyrrolidine on one side forms Pi‐Cat interaction (red line) with H121. The docking was performed by rosetta‐highresdocker with ‘‐nstruct 50 000’ and the left side of the drawing with pymol (https://www.pymol.org) and the right side with maestro (https://www.schrodinger.com/platform/products/maestro).

The GGVS…H (this histidine residue is labeled blue) sequence, which is partially present, as shown in Fig. 1, may be involved in the binding of T2 or T3 copper ions. The QXH…ADCXPVV…HAGWRG sequence can be affirmed as a T1 Cu‐binding site, where the H…C…H pattern matches the T1 Cu‐binding site of a typical laccase. EV sequences in some proteins may form the binding site of a T2 or T3 copper ion with a His residue, which does not show any identity to sequences from other species in this family. The FY(YF/FF)SXRR(L) motif of all proteins belonging to this group is involved in the generation of the substrate pocket and, as shown in the docking results in Fig. 2, the Arg residue of some of the proteins may be involved in substrate binding. As shown in Fig. 1, the conserved sequences of the proteins used in this study possess a high degree of overlap with the sequences of DSM5159 and PsLac, as well as the conserved sequences of the DUF152 family, and especially in the H…C…H amino acid pair of the copper ion binding site. Considering that prokka 1.12.0 (x3) annotation of these sequences resulted in laccase, it is also possible that purine nucleoside phosphorylase has the ability to catalyze laccase substrates. These sequences were subjected to structure prediction and docking.

Many of the sequences annotated as laccase were also annotated as YfiH and DUF152. All enzymes in this subfamily possess a Cys‐His pair. [45] This is similar to the copper‐ion‐binding site of laccase. DUF152 is classified as a subfamily of laccase in some cases. [4] Based on the sequence alignment, the conserved sequence of LMCO used in this study is essentially the same as the conserved sequence of the DUF152 family. Therefore, the LMCOs in this study should belong to the DUF152 family.

### Prediction of three‐dimensional structure

**Fig. 3:**
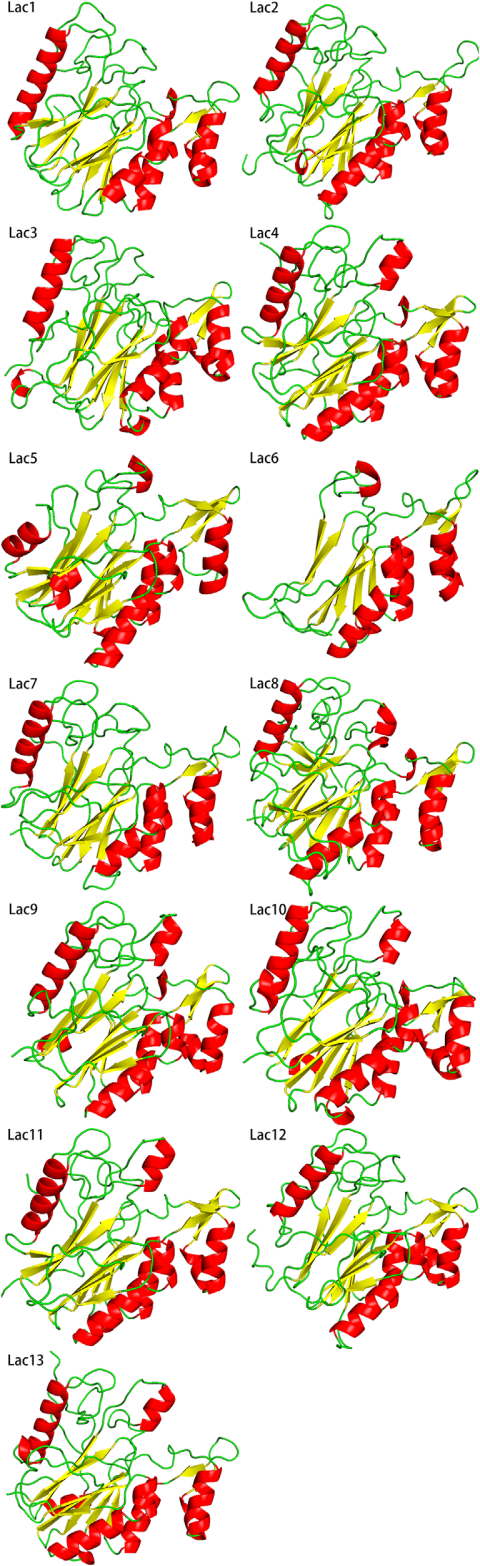
3D structures of the LMCOs (Lac1 to Lac13) predicted by alphafold2. Even though all these LMCOs exhibit low amino acid sequence identity, the overall structures still show a high degree of similarity. The central β‐sheets of all proteins overlap significantly, and the surrounding helices and overall structural trends remain largely consistent. All structures are predicted by alphafold2 with ‘‐model_preset=mono_casp14’.

The three‐dimensional structure of this bacterial LMCO group was predicted from amino acid sequences using alphafold2. As shown in Fig. 3, the main body of the 3D structure of this group exhibits a high degree of overlap. This result is consistent with the fact that, despite the low amino acid sequence identity between these LMCOs, they have similar molecular structures, and the overall geometry of their active sites is highly conserved. [5] This provides a sufficient basis for screening LMCOs based on three‐dimensional structure.

Because of the advantages of fungal laccases for various applications, bacterial laccases and MCOs, especially those with a low molecular mass, have remained relatively underexplored. The RCSB PDB database (https://www.rcsb.org) was searched separately for enzymes belonging to the MCO class, as shown in Table 3. The number of results decreased dramatically when additional search terms for bacteria and molecular masses under 30 kDa were applied. However, the molecular masses of the MCOs used in this study are in the range of 24 to 28.5 kDa. Both the search for similar sequences by Uniprot blast, as shown in Table 1, and the search for similar structures by PDB failed to find the identified MCOs. This highlights the difficulty in obtaining sufficient information from existing experimental results and databases. Structural prediction provides more structural information. It provides the possibility for subsequent docking.

**Table 3:** Search results in the RCSB PDB database.

	All	Bacteria	< 30 kDa
Laccase (EC1.10.3.2)	194	101	0
Ascorbate oxidase (EC1.10.3.3)	4	0	0
Ferroxidase (EC1.16.3.1)	427	126	30
Nitrite reductase (EC1.7.2.1)	298	297	0

### Docking of ABTS

**Fig. 4:**
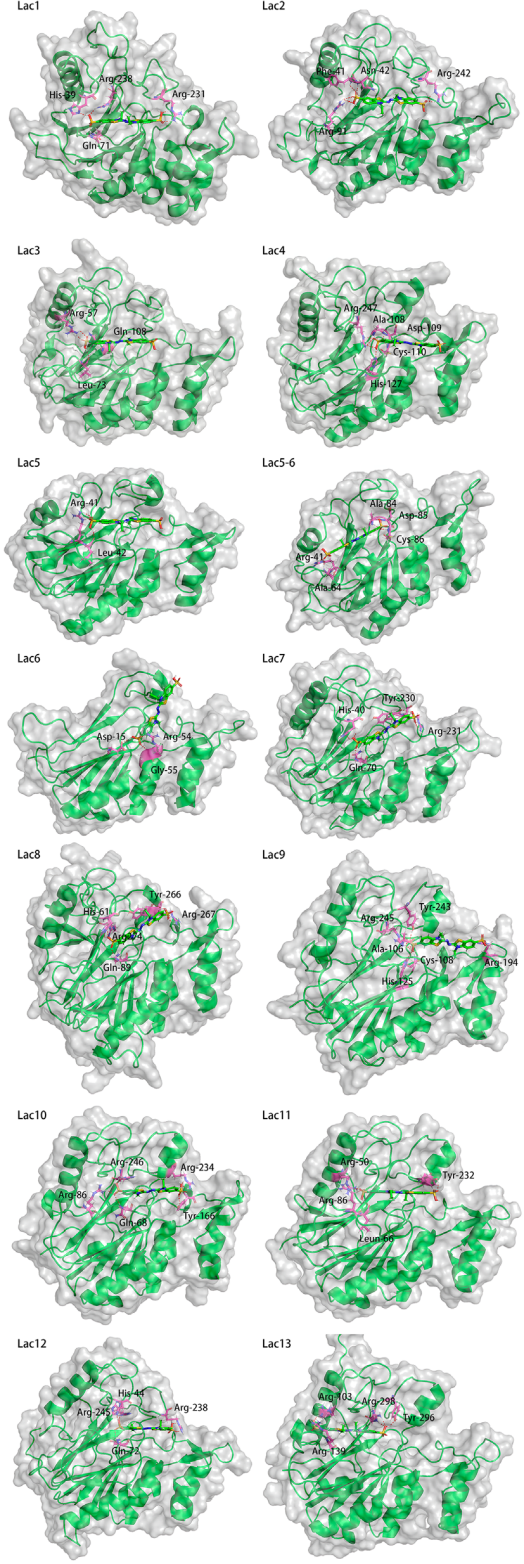
Docking results of Lac1 to Lac13 and Lac5‐6 with ABTS. Predicted hydrogen bonds are indicated using red dashed lines. Amino acid residues that may form hydrogen bonds with ABTS are indicated in purple. The docking was performed using rosetta‐highresdocker with ‘‐nstruct 50 000’ and pymol was used for drawing.

Primary screening by autodock‐vina and rosetta‐highresdocker revealed the following results:

1. All sequences shown in Fig. 4 exhibit distinct substrate pockets.
2. The substrate pockets of all the enzymes overlap with the T1 copper ion position. This also proves the correctness of the simulation by metal3d of T1 copper ions because laccase‐like oxidation reactions require the substrate to lose electrons near the T1 copper ion. Therefore, the T1 copper ion should be located in the same region as the substrate‐binding site.
3. Amino acids are involved in the direct construction of the substrate pocket at approximately every 20 amino acids starting at the N‐terminal ~30th amino acid.
4. Compared to a typical laccase, this type of LMCO, with a full length of about 270 amino acids, is relatively short, and its integrity is critical for construction of the substrate pocket as well as for enzyme activity.
5. As shown in Figs 1 and 2, some of the proteins showed that ABTS may form a hydrogen bond with the first R in the amino acid sequence of FY(YF/FF)SXRR(L) at the C‐terminal end, which provides an idea for the design of new LMCOs to improve the affinity for substrates such as ABTS.

### Docking of the copper ion

**Fig. 5:**
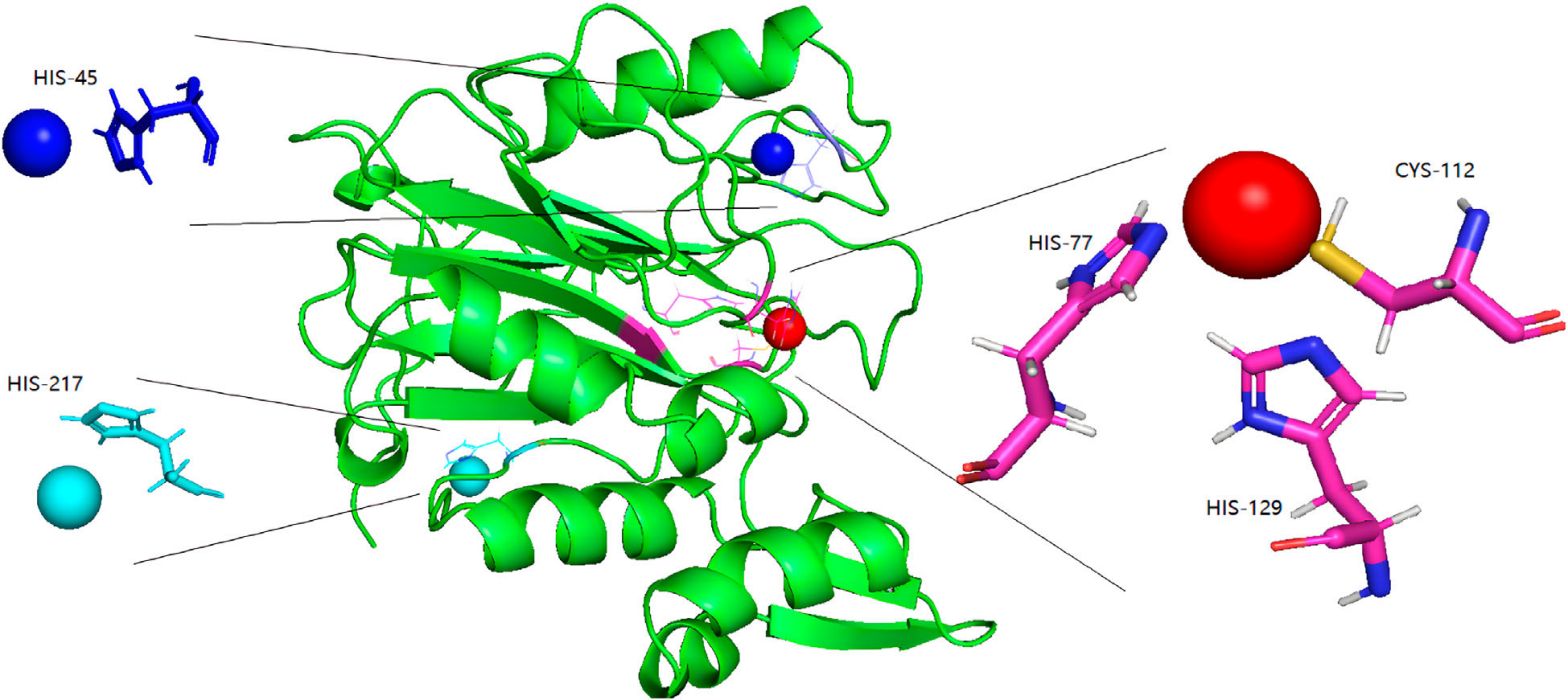
Result of Lac3–Cu docking; red color indicates the T1 copper ion. The docking was performed using metal3d with default parameters and and pymol was used for drawing.

Predictions of copper binding obtained from metal3d demonstrated the following results:

1. The overall copper binding varies greatly from sequence to sequence.
2. Most of the sequences have one copper ion with a confidence metric greater than 0.85, the position of which overlaps with the substrate‐binding pocket, thus corresponding to the T1 copper ion.
3. The binding sites of the T1 copper ion are his near N‐terminal 70, Cys near N‐terminal 110, and his near N‐terminal 125. As shown in Fig. 5, Lac3 serves as a representative example.

### Selection of the LMCO sequences

The simulation results are presented in Table 4. Because there are four copper ion ligands present in a typical laccase molecule, the scoring against copper ion ligands is divided into two parts: the confidence metric of copper ion binding at the optimal position and the average binding confidence metric of the four copper ions. Sequences that showed significant discrepancies between metal3d and autodock‐vina results in terms of copper ion docking and a large gap between rosetta and autodock‐vina results in terms of substrate docking were eliminated. For copper ion docking, the results of metal3d were used. For substrate docking, the results of rosetta were used. Ten sequences were randomly selected that met the following conditions.

1. The confidence metric of copper ion binding at the best position is greater than 0.85.
2. The average copper ion binding confidence metric is greater than 0.2.
3. Interface_delta_X is less than −16.

**Table 4:** Simulated scores for selected sequences.

Name	Numbers of NCBI	Predicted source organisms	Cu1	Cu2	Cu3	Cu4	Cu average	Cu max	Interface_delta_X
Lac1	PP987282	*Acinetobacter venetianus*	0.97	0.36	0	0	0.333	0.97	−19.753
Lac2^a^	PP987283	*Candidimonas bauzanensis*	0.56	0.41	0.29	0	0.315	0.56	−19.720
Lac3	PP987284	*Acidihalobacter prosperus*	0.93	0.28	0.12	0	0.333	0.93	−19.653
Lac4	PP987285	*Novosphingobium*	0.93	0	0	0	0.233	0.93	−16.800
Lac5	PP987286	*Variovorax*	0.88	0.43	0.21	0	0.380	0.88	−17.486
Lac5‐6	–		0.95	0.27	0.12	0	0.335	0.95	−19.776
Lac6^a^	PP987287	*Sphingopyxis indica*	0.77	0	0	0	0.193	0.77	−7.160
Lac7	PP987288	*Pseudomonas balearica*	0.88	0.42	0.21	0	0.378	0.88	−19.397
Lac8	PP987289	*Pusillimonas noertemannii*	0.96	0.5	0.62	0	0.520	0.96	−18.800
Lac9	PP987290	*Rhodospirillaceae*	0.94	0	0	0	0.235	0.94	−20.065
Lac10	PP987291	*Tistlia consotensis*	0.95	0.56	0.27	0.28	0.515	0.95	−18.146
Lac11^a^	PP987292	*Sphingopyxis*	0.43	0.22	0	0	0.163	0.43	−20.178
Lac12	PP987293	Other	0.87	0.57	0.47	0.24	0.538	0.87	−17.190
Lac13	PP987294	*Pedosphaera sp. Tous‐C6FEB*	0.96	0.28	0.42	0	0.415	0.96	−21.698

^a^ Negative sample.

At the same time, two sequences were randomly selected from those that did not meet the above conditions, and the sequence Lac6, which had an obvious deletion at the N‐terminus, was used as a negative control. All sequences were uploaded to NCBI, as shown in Table 4. The N‐terminal end of Lac5 was selected for splicing with Lac6 after computer simulation, and the resulting construct was named Lac5‐6. Their DNA fragments were obtained using fusion PCR.

### Measurement of enzyme activity

As shown in Table 5, all high‐scoring sequences demonstrated varying degrees of enzymatic activity, whereas the low‐scoring sequences exhibited no activity. This suggests a strong correlation between computational scoring and actual enzyme activity. Lac5‐6 exhibited superior activity compared to both original proteins, aligning with the predicted data. This indicates the effectiveness of computational predictions in identifying sequences with enhanced enzymatic performance. The nickel affinity chromatography column employed for the purification of these LMCOs exhibited a noticeable decrease in color and, upon subsequent use, the purification efficacy decreased significantly. It is highly probable that these LMCOs acquired some nickel ions from the affinity column during the purification process, thus resulting in the loss of enzyme activity. The enzyme was shown to have the strong property of binding other divalent metal ions. Lac1 and Lac3 exhibited reduced enzymatic activity in reaction systems lacking added copper ions. This may have been caused by loss of copper ions during the purification process.

**Table 5:** Enzyme activity measurements. Activity assay was performed using purified enzyme at a final concentration of 100 mm acetate buffer with 2.5 mm copper sulfate, using ABTS as the substrate.

	pH	Temperature (°C)	*K*_m_ (μm)	*K*_cat_ (min^−1^)	*K*_cat_/*K*_m_ (m ^−1^·s^−1^)
Lac1	5.0	55	1.12 × 10^2^ ± 3.3	0.89 × 10^3^ ± 8.3	1.33 × 10^5^
Lac2	No activity				
Lac3	4.6	60	0.99 × 10^2^ ± 0.4	1.11 × 10^3^ ± 7.5	1.86 × 10^5^
Lac4	4.0	55	4.26 × 10^2^ ± 16.2	0.90 × 10^3^ ± 13.7	0.35 × 10^5^
Lac5	4.0	50	0.63 × 10^2^ ± 0.2	0.81 × 10^3^ ± 15.2	2.14 × 10^5^
Lac5‐6	4.0	55	0.55 × 10^2^ ± 0.4	1.02 × 10^3^ ± 7.2	3.06 × 10^5^
Lac6	No activity				
Lac7	4.8	45	2.31 × 10^2^ ± 1.1	0.85 × 10^3^ ± 13.6	0.61 × 10^5^
Lac8	No activity after purification				
Lac9	4.8	55	1.01 × 10^2^ ± 0.6	0.95 × 10^3^ ± 10.3	1.57 × 10^5^
Lac10	4.4	60	1.05 × 10^2^ ± 0.6	0.94 × 10^3^ ± 14.1	1.49 × 10^5^
Lac11	No activity				
Lac12	5.0	55	1.11 × 10^2^ ± 1.4	0.65 × 10^3^ ± 10.6	0.98 × 10^5^
Lac13	No activity after purification				

Nine of the proteins obtained showed superior activity under acidic conditions. This group of proteins showed little difference between ABTS and protein docking results, as shown in Table 4. The binding of T1 copper ions to the proteins emerged as a crucial factor influencing enzyme activity. This parameter should be prioritized as the primary criterion for LMCO selection.

**Fig. 6:**
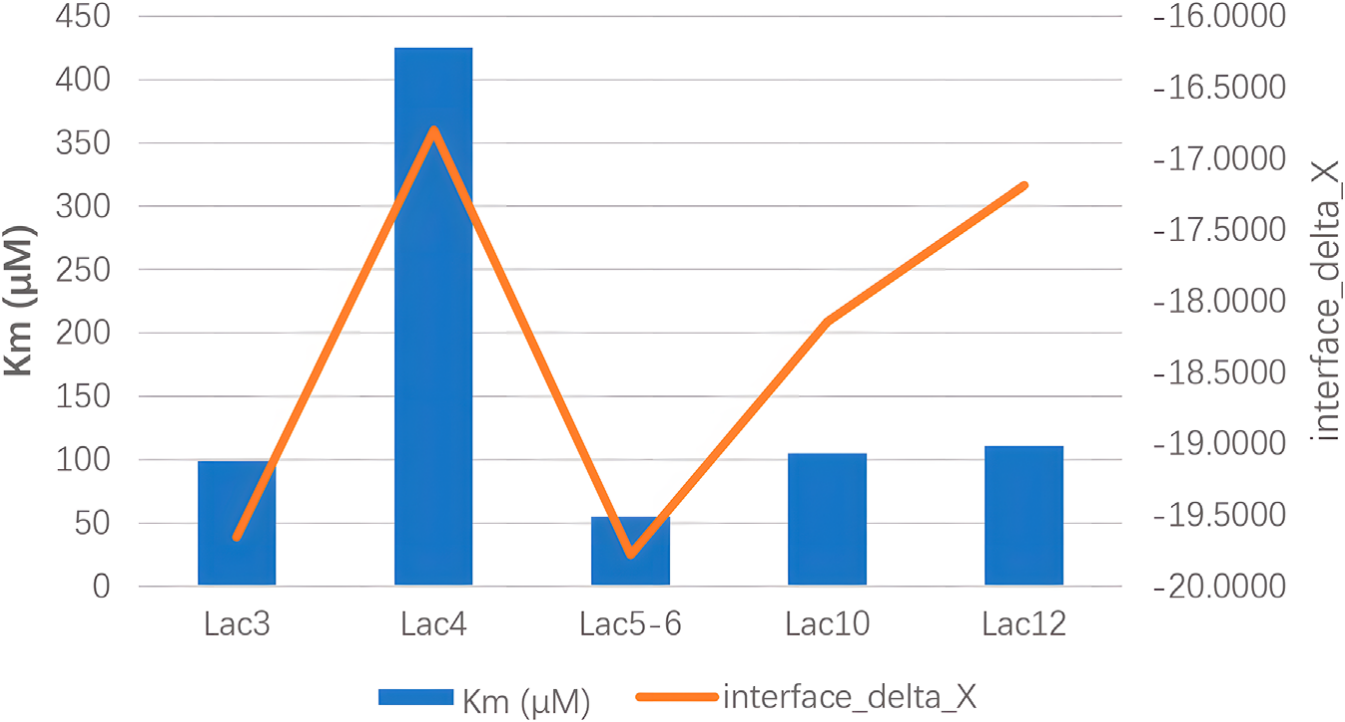
Substrate docking results with *K*_m_; docking results in orange and *K*_m_ in blue. The data for interface_delta_X come from the docking by rosetta‐highresdocker with ‘‐nstruct 50 000’. The data for *K*_m_ are the average of three measurements.

In addition, numerous other factors affect laccase activity, such as the binding of copper ions, electron transport and substrate binding, amongst many more. The activities of nine MCOs were measured: five MCOs showed marked correlation between the substrate docking results and *K*_m_, with a correlation coefficient of 0.69, as shown in Fig. 6. When all nine MCOs were considered, the correlation coefficient was still 0.45. This can also be used to screen laccases for potential activity against a particular substrate.

## Concluding remarks

In the present study, we retrieved 34 possible bacterial LMCOs from industrial wastewater metagenome sequences. A set of 10 novel LMCOs was identified using information technology to predict their three‐dimensional structure, copper binding and substrate binding, as well as to repair one sequence‐deficient protein. This group of LMCOs did not show any sequence similarities to previously reviewed laccases or MCOs, and they possessed optimal activity in an acidic environment. The results of the enzyme activity measurements were consistent with those obtained from computer simulations. Finally, this study demonstrates the value of combining metagenomic sequencing technology with protein structure modeling in the search for novel enzymes, as well as in the screening and design of novel enzymes. The combination of several types of protein structure simulation software can automate the process from metagenome‐derived sequences to the prediction of enzyme activity and specificity.

## Conflicts of interest

The authors declare that they have no conflicts of interest.

## Peer review

The peer review history for this article is available at https://www.webofscience.com/api/gateway/wos/peer‐review/10.1002/2211‐5463.70037.

## Author contributions

AK and HB provided DNA samples. AP and RD completed the Metagenomic sequencing. TC and H‐JM conceived the study. H‐JM provided equipment and funding for sequence screening, computer simulation and gene expression. TC performed the sequence screening, computer simulation, gene expression and wrote the original draft. All authors have contributed to editing the mnuscript.

## Data Availability

The data that support the findings of this study are available from the corresponding author upon reasonable request. Some of the data has been submitted to the Zenodo repository (https://doi.org/10.5281/zenodo.15123719).
